# Prevalence, awareness, treatment, control and risk factors related to hypertension among urban adults in Inner Mongolia 2014: differences between Mongolian and Han populations

**DOI:** 10.1186/s12889-016-2965-5

**Published:** 2016-04-01

**Authors:** Guoju Li, Hailing Wang, Ke Wang, Wenrui Wang, Fen Dong, Yonggang Qian, Haiying Gong, Guodong Xu, Yanlong Li, Li Pan, Bin Wang, Guangjin Zhu, Guangliang Shan

**Affiliations:** Department of Epidemiology and Statistics, Institute of Basic Medical Sciences, Chinese Academy of Medical Sciences, School of Basic Medicine, Peking Union Medical College, Beijing, 100005 China; Inner Mongolia Center for Disease Control and Prevention, Hohhot, Inner Mongolia Autonomous Region 010110 China; Department of Epidemiology and Health Statistics, College of Public Health, Jilin University, Changchun, 130021 China

**Keywords:** Hypertension, Mongolian, Han, Urban, Ethnicity

## Abstract

**Background:**

Han and Mongolian populations constitute approximately 96 % of the population of Inner Mongolia Autonomous Region, and the two ethnic groups have different genetic backgrounds and lifestyle. We aim to assess the prevalence, awareness, treatment, control, and related risk factors of hypertension among urban adults in Inner Mongolia, with the comparison of the differences between Mongolian and Han populations in this respect.

**Methods:**

Three thousand two hundred fifty-one individuals aged 20–80 years (2326 Han and 925 Mongolian) were selected using a multistage cluster sampling method from Inner Mongolia in 2014. The adjusted prevalence, awareness, treatment and control of hypertension were evaluated by the Logistic regression. In addition, possible interactions were also tested. When interactions were found significant, strata-specific analysis were performed. Multivariate logistic regression was used for estimating independent associations between risk factors and hypertension.

**Results:**

The prevalence of hypertension was 27.47 % for Han population, 31.46 % for Mongolian population. The adjusted prevalence, awareness, treatment and control of hypertension were 26.45, 65.43, 78.24 and 48.28 % in Han, and 31.30, 68.22, 85.57 and 50.55 % in Mongolian, respectively. There was no significant difference in the adjusted awareness, treatment and control of hypertension among Mongolian and Han adult residents (all *P* >0.05). Lower prevalence of hypertension was associated with younger age and healthy weight in both Mongolian and Han adults. Within Han adults, high education, moderate physical activity and non-alcohol drinkers were additionally associated with lower prevalence of hypertension, whereas within Mongolian adults, lower prevalence was associated with being female. Among residents with medium education level, nondrinkers had 0.60 times lower odds of having hypertension than current drinkers (OR = 0.60, 95 % CI: 0.44–0.82); among residents with high education level, nondrinkers has 0.65 times lower odds of having hypertension than current drinkers (OR = 0.65, 95 % CI: 0.43–0.97).

**Conclusions:**

Mongolian population had a higher prevalence of hypertension than Han population. There were no significant difference between Mongolian and Han population in awareness, treatment and control of hypertension, which suggested that there was no difference between the two ethnicities in the distribution of health resources.

## Background

Hypertension is one of the most important risk factors for mortality worldwide, and is common in low- and middle-income countries [1]. It is interrelated with coronary artery disease, stroke, congestive heart failure, and renal dysfunction. Hypertension is also a major cause of disability and is considered to be the leading cause for death in the world [2]. In the INTERHEART study, 66 % of stroke was attributable to hypertension and 17–22 % of acute myocardial infarction in South Asian and Chinese populations was attributable to hypertension [3, 4]. It is estimated that in China 230 million people suffer from cardiovascular diseases, out of which 200 million have hypertension [5]. Several studies in developed countries have shown an increasing trend in the prevalence of hypertension [6, 7], while many studies also indicated an increasing trend in hypertension prevalence in China [8–11]. The prevalence of hypertension had been noted to be higher in northern regions compared with those in southern regions and there were marked ethnical difference in blood pressure level and the prevalence of hypertension in China. The Inner Mongolia Autonomous Region is located in northern China, and Han and Mongolian constitute approximately 96 % of the total population. The two ethnic groups have different genetic backgrounds, culture, customs and food consumption. Mongolian tend to eat more animal fat, drink strong wine, consume less grain, fresh vegetables, beans, and unsaturated fatty acids [12]. One objective of the study is to provide updated finding in prevalence, awareness, treatment, and control of hypertension between Han and Mongolian populations and to assess risk factors associated with hypertension in the two ethnic groups.

## Methods

The China National health survey (CNHS) was conducted by the Chinese Academy of Medical Sciences for evaluating the Physiological Constant and Health Condition in Chinese. The part we reported in this study was conducted in Inner Mongolia Autonomous Region in 2014. A sample of adult residents aged 20–80 years was selected using a multistage cluster sampling method. The sampling process was stratified according to degree of urbanization, and four urban areas were selected from Inner Mongolia Autonomous Region including Bayan Nur, Xilingol League, Ulanqab and Hohhot. In each area, different districts were selected as sampling units. Participants recruited including residents who had been living in Inner Mongolia for more than 1 year. Data on demographic information, smoking habit, alcohol drinking habit and history of hypertension were collected using unified questionnaire for all participants. In the meantime, body height and weight were measured with subjects to be barefoot. Height was measured to the nearest 0.1 cm using a fixed stadiometer and weight was measured to the nearest 0.01 km by BIA (bioelectrical impendence analysis) with a commercially available body composition analyzer (BC-420, TANITA, Japan). Body mass index (BMI) was defined as weight/height^2^ (kg/m^2^). All investigators were trained before the survey, and all participants provided written informed consent. The study was approved by the Institutional Review Board of the Institute of Basic Medical Sciences, Chinese Academy of Medical Sciences.

### Definitions

Ethnicity was determined by the Subjects’ ID card and categorized as Mongolian and Han people. Subjects were considered to be Mongolian or Han people if they and their parents were with the same ethnicity.

BMI was categorized according to the World Health Organization criteria, with BMI < 25 kg/m^2^ defined as lean or healthy, BMI between 25 and 29 kg/m^2^ defined as overweight, and BMI ≥ 30 kg/m^2^ defined as obese.

Education was categorized as low (received only primary education or no education), medium (finished secondary school or high school) and high (graduated from college or university).

Current smokers were defined as those who smoked at least one cigarette per day and had lasted for at least 6 months. Ex-smokers were defined as those who had stopped smoking more than 6 months prior to the study.

Current drinkers were defined as those who drank at least twice per month (more than 640 ml beer or 100 ml Chinese liquor, about 57 g alcohol), and had lasted for at least 6 months. Ex-drinkers were defined as those who had stopped drinking more than 6 months prior to the study.

Physical activity was divided into three categories: (1) light: e.g., office worker, salesperson, and house worker; (2) Medium: e.g., driver, electrician, and latheman; (3) Heavy: e.g., manual worker, steel worker, and mineworker.

Exercise was divided into two categories: (1) exercise: regular exercise at least once a week (e.g., fast walks, skiing, swimming, jogging, cycling, running, ball games) and lasted at least 30 min; (2) no exercise: practically no exercise at all.

Sitting blood pressure was measured by trained research assistants following a standardized procedure using Omron digital blood pressure measuring device (HEM907). Cuff sizes were chosen based on the participants’ arm circumference. Participants were advised to avoid cigarette smoking, alcohol, caffeinated beverages, and exercise for at least 30 min before measurement.

Hypertension was defined as an average (calculated from three measurements) systolic blood pressure (SBP) ≥140 mmHg, or an average diastolic blood pressure (DBP) ≥90 mmHg, or self-reported diagnosis of hypertension.

Awareness of hypertension was defined as the proportion of people with hypertension who reported a medical diagnosis.

Treatment of hypertension was defined as the self-reported use of pharmacological medication for the high Blood pressure.

Among hypertensive participants who were under treatment, control was defined as the proportion of the individuals who had an average systolic and diastolic blood pressure of less than 140/90 mmHg.

### Statistical analysis

Results are presented as the percentage and 95 % confidence interval (CI) in each subgroup for categorical variables and mean ± SD for continuous variables. Chi-square test and *t*-test were used to examine differences in variables between Mongolian and Han populations. The adjusted prevalence, proportion of awareness, treatment and control of hypertension were evaluated by the Logistic regression [13]. Interaction between variables was tested by the multivariate logistic regression models. When interactions were found significant, strata-specific analysis were performed for adjusted rates. Multivariate logistic regression analysis was used for estimating independent association between risk factors and hypertension. All tests were two-sided with significance level of 0.05. All of the analyses were performed using SAS software version 9.2 (SAS Institute Inc., Cary, NC, USA).

## Results

In the present study, a total of 3251 individuals were included in the analysis, of which 925 (28.45 %) were Mongolian. The general characteristics associated with hypertension among Han and Mongolian are shown in Table 1. When compared to the Han sample, the Mongolian sample were more likely to be highly educated, overweight or obese, current alcohol drinkers, undertaking moderate or high physical activity and doing exercise (*P* < 0.05). For both Han and Mongolian, overweight and obesity rate, current smoking, current alcohol drinking, undertaking moderate or high physical activity appeared to be more common in male than in female (*P* < 0.05). For Han people, the percentage of high education level and exercise were higher in male than that in female (*P* < 0.05).

**Table 1:** Demographic characteristic of the study population

Characteristic	Han (*n* = 2326)	Mongolian (*n* = 925)	All (*n* = 3251)	*P* value
Male, %	38.65 (36.67–40.63)	38.81 (35.67–41.95)	38.7 (37.02–40.37)	0.93
Mean age, yrs	45.00 ± 13.66	44.43 ± 13.47	44.84 ± 13.60	0.28
Age groups, %				
20–29	16.98 (15.45–18.51)	18.38 (15.88–20.88)	17.38 (16.08–18.68)	
30–39	17.28 (15.74–18.82)	18.16 (15.68–20.64)	17.53 (16.22–18.84)	
40–49	29.02 (27.18–30.86)	27.68 (24.80–30.56)	28.64 (27.09–30.19)	
50–59	20.98 (19.33–22.63)	21.62 (18.97–24.27)	21.16 (19.76–22.56)	
60–80	15.74 (14.26–17.22)	14.16 (11.91–16.41)	15.29 (14.05–16.53)	0.62
Education level, %				
Low	13.46 (12.07–14.85)	11.24 (9.20–13.28)	12.83 (11.68–13.98)	
Medium	54.08 (52.05–56.11)	37.3 (34.18–40.42)	49.31 (47.59–51.03)	
High	32.46 (30.56–34.36)	51.46 (48.24–54.68)	37.87 (36.20–39.54)	<0.001
Cigarette smoking, %				
Never	69.69 (67.82–71.56)	69.51 (66.54–72.48)	69.64 (68.06–71.22)	
Current	22.91 (21.20–24.62)	21.84 (19.18–24.50)	22.61 (21.17–24.05)	
Former	7.39 (6.33–8.45)	8.65 (6.84–10.46)	7.75 (6.83–8.67)	0.43
Alcohol drinking, %				
Never	59.24 (57.24–61.24)	49.73 (46.51–52.95)	56.54 (54.84–58.24)	
Current	34.74 (32.80–36.68)	42.81 (39.62–46.00)	37.03 (35.37–38.69)	
Former	6.02 (5.05–6.99)	7.46 (5.77–9.15)	6.43 (5.59–7.27)	<0.001
BMI, %				
Lean or healthy	58.00 (55.99–60.01)	49.95 (46.73–53.17)	55.71 (54.00–57.42)	
Overweight	33.71 (31.79–35.63)	40.43 (37.27–43.59)	35.62 (33.97–37.27)	
Obesity	8.30 (7.18–9.42)	9.62 (7.72–11.52)	8.67 (7.70–9.64)	<0.001
Physical activity, %				
Light	80.91 (79.31–82.51)	82.38 (79.92–84.84)	81.33 (79.99–82.67)	
Medium	12.77 (11.41–14.13)	8.86 (7.03–10.69)	11.66 (10.56–12.76)	
Heavy	6.32 (5.33–7.31)	8.76 (6.94–10.58)	7.01 (6.13–7.89)	<0.001
Exercise, %	57.52 (55.51–59.53)	62.92 (59.81–66.03)	59.06 (57.37–60.75)	0.005

Overall, the prevalence of hypertension was 28.61 % (27.47 % for Han people, 31.46 % for Mongolian people). In Table 2, compared to the Han sample, the prevalence of hypertension in Mongolia sample was significantly higher in male and in the population aged 50 or over, never smoking and with medium or high education (*P* < 0.05).

**Table 2:** Prevalence of hypertension in Han and Mongolian adults

Characteristic	Han (*n* = 2326)	Mongolian (*n* = 925)	All (*n* = 3251)	*P* value
All, %	27.47 (25.66–29.29)	31.46 (28.47–34.45)	28.61 (27.05–30.16)	0.02
Sex, %				
Male	34.04 (30.94–37.14)	40.67 (35.59–45.75)	34.18 (31.56–36.80)	0.03
Female	23.34 (21.14–25.53)	25.62 (22.02–29.21)	23.98 (22.11–25.86)	0.28
Age groups, %				
20–29	3.29 (1.53–5.05)	5.29 (1.93–8.66)	3.89 (2.30–5.49)	0.26
30–39	13.68 (10.32–17.04)	11.31 (6.52–16.10)	12.98 (10.22–15.74)	0.44
40–49	26.67 (23.33–30.00)	28.91 (23.35–34.46)	27.28 (24.42–30.14)	0.49
50–59	40.57 (36.22–44.93)	50.00 (43.07–56.93)	43.31 (39.61–47.02)	0.02
60–80	52.73 (47.62–57.85)	67.94 (59.95–75.93)	56.74 (52.38–61.10)	0.003
Education level, %				
Low	41.53 (36.07–46.99)	48.08 (38.48–57.68)	43.17 (38.41–47.92)	0.24
Medium	29.81 (27.28–32.34)	37.97 (32.85–43.09)	31.57 (29.29–33.84)	0.004
High	17.75 (15.02–20.47)	23.11 (19.32–26.90)	19.82 (17.59–22.05)	0.02
Cigarette smoking, %				
Never	24.00 (21.92–26.08)	28.62 (25.12–32.11)	25.31 (23.52–27.10)	0.02
Current	30.21 (26.31–34.10)	30.69 (24.33–37.05)	30.34 (27.02–33.66)	0.90
Former	51.74 (44.28–59.21)	56.25 (45.38–67.12)	53.17 (47.01–59.34)	0.50
Alcohol drinking, %				
Never	24.75 (22.47–27.02)	27.83 (23.73–31.92)	25.52 (23.52–27.51)	0.19
Current	29.95 (26.79–33.11)	32.58 (27.96–37.19)	30.81 (28.21–33.42)	0.35
Former	40.00 (31.88–48.12)	49.28 (37.48–61.07)	43.06 (36.35–49.78)	0.20
BMI, %				
Lean or healthy	17.27 (15.25–19.29)	17.75 (14.26–21.23)	17.39 (15.65–19.14)	0.82
Overweight	38.39 (34.99–41.80)	42.25 (37.24–47.25)	39.64 (36.82–42.45)	0.21
Obesity	54.40 (47.38–61.43)	57.30 (47.03–67.58)	55.32 (49.52–61.12)	0.65
Physical activity, %				
Light	28.27 (26.23–30.30)	31.89 (28.58–35.20)	29.31 (27.58–31.05)	0.06
Medium	21.89 (17.18–26.59)	26.83 (17.24–36.42)	22.96 (18.72–27.19)	0.35
Heavy	28.57 (21.27–35.87)	32.10 (21.93–42.27)	29.82 (23.89–35.76)	0.58
Exercise, %				
Yes	32.66 (30.15–35.17)	36.43 (32.52–40.34)	33.80 (31.69–35.92)	0.11
No	20.45 (17.93–22.96)	23.03 (18.58–27.49)	21.11 (18.92–23.30)	0.31

The prevalence of awareness, treatment and control of hypertension are presented in Table 3. The majority of the sample (66.34 %) were aware of hypertension as a disease. Among them, 87.03 % had ever received some kind of antihypertensive therapy, and 43.58 % of those ever taking antihypertensive therapy now has normal blood pressure. Compared to male, the awareness, treatment and control were higher in female (*P* <0.05). The awareness of hypertension increased with age. In addition, the awareness was higher among non alcohol drinkers or former alcohol drinkers than current alcohol drinkers, and higher in population with low education and exercise than otherwise. The proportion of treatment was higher in the population with heavy physical activity and exercise. The proportion of control was higher in non-alcohol drinkers or former alcohol drinkers and in the population with low education (*P* <0.05).

**Table 3:** Awareness, treatment and control of hypertension in the study population

Variable	Total number of people with hypertension	Awareness	Treatment	Control
		% (95 % CI)	% (95 % CI)	% (95 % CI)
All	930	66.34 (63.31–69.38)	87.03 (84.38–89.68)	43.58 (39.38–47.77)
Sex: Male (reference)	452	60.62 (56.12–65.12)	83.94 (79.59–88.29)	38.70 (32.40–44.99)
Female	478	71.76 (67.72–75.79) ^*^	89.50 (86.26–92.75)**	47.23 (41.65–52.82)**
Ethnic groups: Mongolian (reference)	291	69.07 (63.76–74.38)	89.05 (84.74–93.37)	43.58 (36.31–50.84)
Han	639	65.10 (61.41–68.80)	86.06 (82.73–89.39)	43.58 (38.44–48.71)
Age Groups, %: 30–39 (reference)	74	40.54 (29.35–51.73)	73.33 (57.51–89.16)	27.27 (8.66–45.88)
20–29	22	27.27 (8.66–45.88)	16.67 (0.00–46.49) *	0.00 (0.00–0.00)
40–49	254	57.48 (51.40–63.56)*	81.51 (75.21–87.80)	43.70 (34.79–52.61)
50–59	298	71.81 (66.70–76.92) *	88.79 (84.56–93.01) **	42.11 (35.08–49.13)
60–80	282	78.37 (73.56–83.17) *	92.76 (89.34–96.18) *	46.83 (40.00–53.66)
Education level,%: Low (reference)	180	76.67 (70.49–82.85)	92.75 (88.43–97.08)	55.47 (46.86–64.08)
Medium	506	67.00 (62.90–71.09) **	85.25 (81.48–89.03)	40.48 (34.83–46.14) *
High	244	57.38 (51.17–63.58) *	85.71 (79.92–91.51)	38.33 (29.63–47.03) *
Cigarette smoking, %: Never (reference)	573	66.49 (62.63–70.36)	88.19 (84.95–91.43)	44.94 (39.62–50.26)
Current	223	60.99 (54.58–67.39)	83.82 (77.63–90.01)	43.86 (34.75–52.97)
Former	134	74.63 (67.26–81.99)	87.00 (80.41–93.59)	37.93 (27.74–48.13)
Alcohol drinking, %: Never (reference)	469	70.79 (66.67–74.90)	89.46 (86.15–92.76)	47.47 (41.80–53.15)
Current	371	58.49 (53.48–63.50) *	82.49 (77.43–87.55) **	36.31 (29.27–43.36)
Former	90	75.56 (66.68–84.43)	89.71 (82.48–96.93)	45.90 (33.40–58.41)
BMI, %: Lean or healthy (reference)	315	60.95 (55.56–66.34)	86.46 (81.62–91.30)	49.40 (41.79–57.00)
Overweight	459	68.85 (64.61–73.08)	87.03 (83.32–90.73)	41.09 (35.28–46.91)
Obesity	156	69.87 (62.67–77.07)	88.07 (81.99–94.16)	40.63 (30.80–50.45)
Physical activity, %: Light (reference)	775	66.32 (63.00–69.65)	87.74 (84.91–90.58)	41.46 (36.92–46.01)
Medium	87	57.47 (47.08–67.86)	78.00 (66.52–89.48)	41.03 (25.59–56.46)
Heavy	68	77.94 (68.09–87.80)	88.68 (80.15–97.21)	65.96 (52.41–79.50) *
Exercise, %: Yes (reference)	649	68.72 (65.15–72.29)	88.34 (85.36–91.32)	44.16 (39.26–49.07)
No	281	60.85 (55.15–66.56) **	83.63 (78.08–89.17) **	41.96 (33.87–50.05)

^*^ *P* < 0.01 (compared with the reference by theχ^2^-test). ^**^ *P* < 0.05 (compared with the reference by theχ^2^-test)

**Fig. 1:**
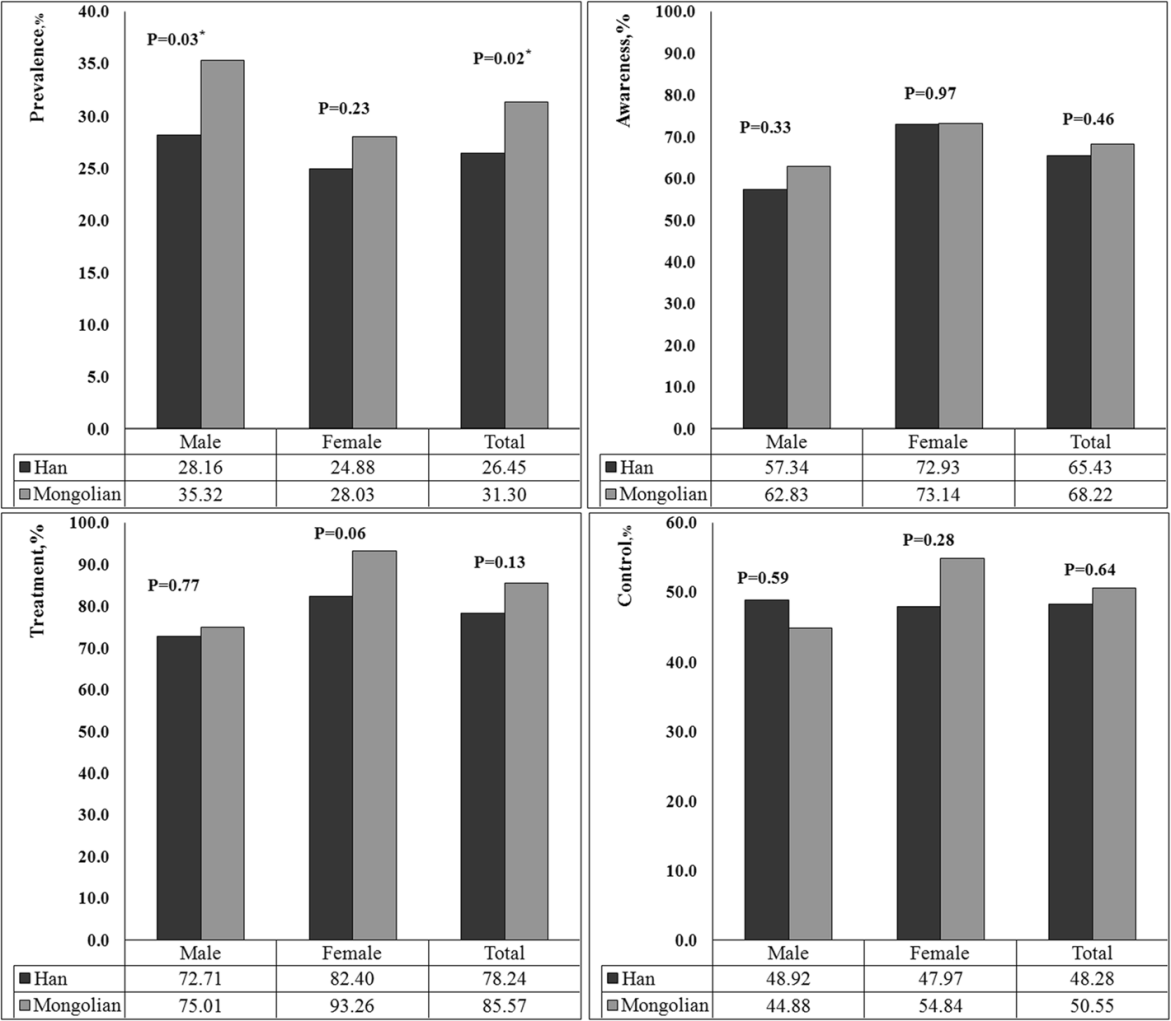
The adjusted prevalence, awareness, treatment and control of hypertension by sex in Han and Mongolian. The prevalence, awareness, treatment and control of hypertension by sex in Han and Mongolian were adjusted for age, body mass index, education, alcohol drinking, cigarette smoking, exercise and activity

Figure 1 shows the adjusted prevalence, awareness, treatment and control of hypertension by sex among Han and Mongolian adult residents. The adjusted prevalence of hypertension was similar with female in both Mongolian and Han (*P* = 0.23). However, the adjusted prevalence among Mongolian males was higher than that among Han males (*P* = 0.03). And the total adjusted prevalence was substantially higher in Mongolian than that in Han (*P* = 0.02). The adjusted awareness, treatment and control of hypertension were 65.43, 78.24 and 48.28 % in Han; and 68.22, 85.57 and 50.55 % in Mongolian, respectively. There was no significant difference in the adjusted awareness, treatment and control of hypertension among Mongolian and Han adult residents (*P* > 0.05).

**Fig. 2:**
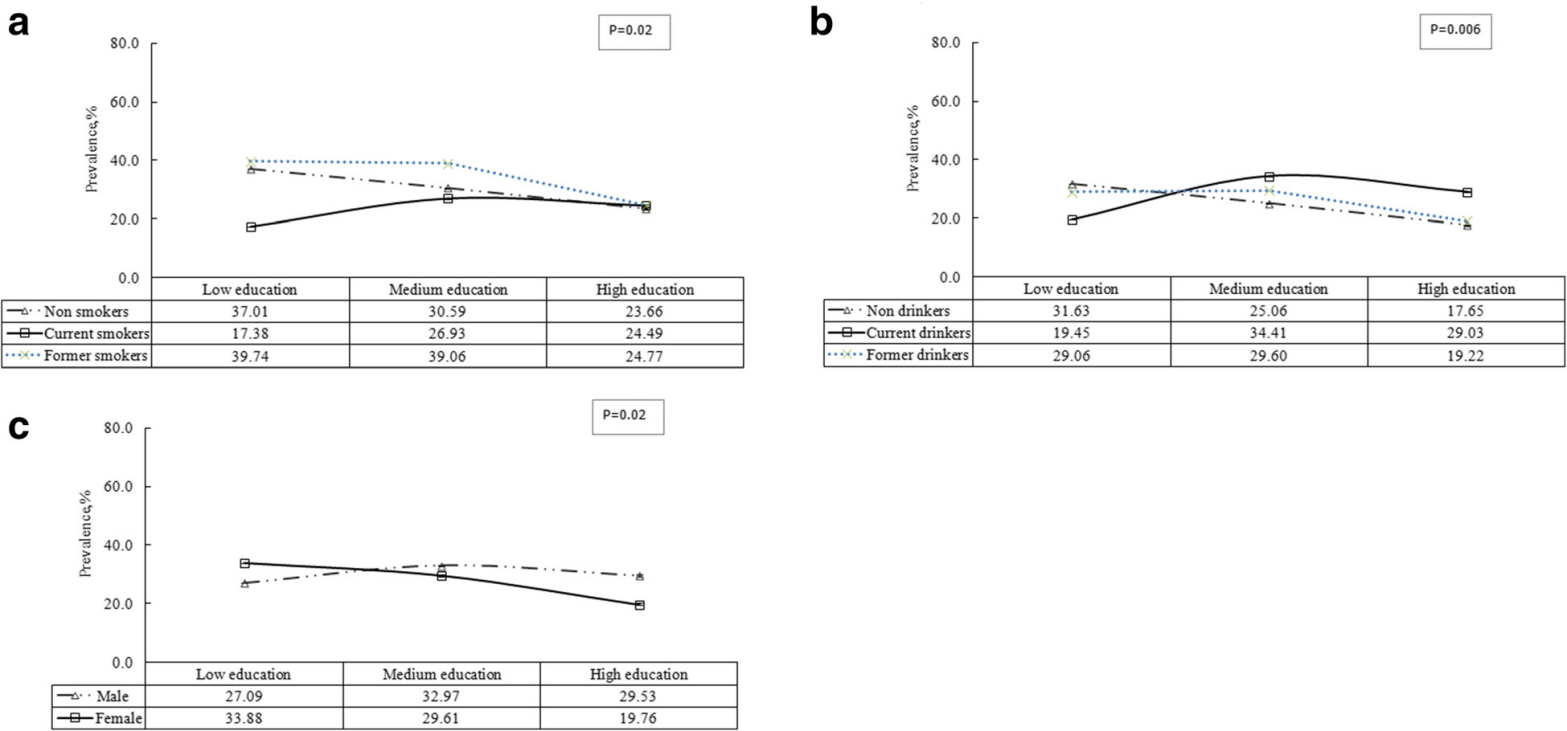
The adjusted prevalence of hypertension by education in sex (**c**), smoking status (**a**) and alcohol drinking status (**b**). The Adjusted prevalence of hypertension by education in the study population were adjusted for sex, ethnicity, age, body mass index, alcohol drinking, cigarette smoking, exercise and activity

We tested the interactions between education and other variables, and there were significant interactions between sex and education (*P* = 0.02), smoking status and education (*P* = 0.02) and alcohol drinking status and education (*P* = 0.006) (Fig. 2). In models mutually adjusted by age, sex, ethnicity, smoking status, alcohol drinking status, BMI, physical activity status and exercise, higher education was associated with lower prevalence of hypertension among females but not males, and higher education was associated with lower prevalence of hypertension among nonsmokers, former smokers, nondrinkers or former drinkers, but not in current smokers or current alcohol drinkers.

Table 4 presents the results of multiple logistic regression analysis of associations between potential risk factors with hypertension in Mongolian and Han. When the multivariate model for the total study contains the interactions of drinking*education and sex*education, the fit of the model is the best. Lower prevalence of hypertension was associated with younger age and healthy weight in both Mongolian and Han adults. Within Han adults, high education, moderate physical activity and non-alcohol drinkers were additionally associated with lower prevalence of hypertension, whereas within Mongolian adults, lower prevalence was associated with being female.

**Table 4:** Factors associated with the prevalence of hypertension in Mongolian and Han people by multivariate logistic regression models

Factors	Mongolian ^a^	*P*	Han ^a^	*P*	Total ^c^	*P*
	Odds ratio (95 % CI)		Odds ratio (95 % CI)		Odds ratio (95 % CI)	
Sex: Male (reference)	1.00		1.00		1.00	
Female	0.55 (0.34–0.88)	0.01	0.74 (0.52–1.06)	0.10	1.21 (0.53–2.73)	0.65
Ethnic groups: Han (reference)	—	—	—	—	1.00	
Mongolian	—	—	—	—	1.27 (1.04–1.55)	0.02
Age groups: 20–29 (reference)	1.00		1.00		1.00	
30–39	1.95 (0.84–4.55)	0.12	3.93 (2.08–7.42)	<0.001	3.46 (2.03–5.90)	<0.001
40–49	5.81 (2.75–12.30)	<0.001	8.36 (4.61–15.15)	<0.001	8.04 (4.90–13.19)	<0.001
50–59	12.88 (5.95–27.89)	<0.001	14.81 (8.11–27.03)	<0.001	15.33 (9.28–25.32)	<0.001
60–80	31.48 (13.23–74.94)	<0.001	22.54 (11.97–42.47)	<0.001	26.24 (15.42–44.65)	<0.001
Education level: Low (reference)	1.00		1.00		1.00	—
Medium	1.15 (0.65–2.02)	0.63	0.83 (0.61–1.11)	0.21	1.25 (0.62–2.51)	0.53
High	0.97 (0.51–1.85)	0.93	0.54 (0.37–0.78)	0.001	1.00 (0.27–3.71)	0.99
Cigarette smoking: Never (reference)	1.00		1.00		1.00	
Current	0.62 (0.38–1.01)	0.06	0.92 (0.65–1.30)	0.64	0.82 (0.62–1.09)	0.17
Former	0.80 (0.43–1.48)	0.47	1.34 (0.87–2.07)	0.19	1.27 (0.89–1.80)	0.19
Alcohol drinking: Never (reference)	1.00		1.00		1.00	–
Current	1.46 (0.95–2.25)	0.09	1.47 (1.11–1.95)	0.007	0.64 (0.27–1.52)	0.31
Former	1.06 (0.55–2.04)	0.86	1.17 (0.75–1.83)	0.48	0.79 (0.46–1.36)	0.39
BMI: Lean or healthy (reference)	1.00		1.00		1.00	
Overweight	2.29 (1.60–3.27)	<0.001	2.30 (1.84–2.86)	<0.001	2.27 (1.87–2.74)	<0.001
Obesity	4.18 (2.42–7.23)	<0.001	5.40 (3.79–7.67)	<0.001	5.02 (3.73–6.75)	<0.001
Physical activity: Light (reference)	1.00		1.00		1.00	
Medium	0.93 (0.50–1.73)	0.81	0.70 (0.50–0.98)	0.04	0.75 (0.55–1.01)	0.06
Heavy	0.90 (0.48–1.68)	0.74	0.64 (0.42–1.00)	0.05	0.78 (0.55–1.11)	0.17
Exercise: No (reference)	1.00		1.00		1.00	
Yes	1.13 (0.77–1.68)	0.53	1.20 (0.95–1.51)	0.12	1.16 (0.95–1.42)	0.13
Sex* Education	—	—	—	—	0.80 (0.57–1.13)	0.21
Alcohol drinking* Education	—	—	—	—	1.21 (1.01–1.46)	0.04

^a^ Adjusted Odds Ratio including sex, age, education, body mass index, alcohol drinking, cigarette smoking, exercise and activity

^c^ Adjusted Odds Ratio including sex, ethnic groups, age, education, body mass index, alcohol drinking, cigarette smoking, exercise, activity, sex*education and alcohol Drinking* Education

There were significant interactions between alcohol drinking status and education (*P* =0.04). Among residents with medium education level, nondrinkers had 0.60 times lower odds of having hypertension than current drinkers (OR = 0.60, 95 % CI: 0.44–0.82); among residents with high education level, nondrinkers has 0.65 times lower odds of having hypertension than current drinkers (OR = 0.65, 95 % CI: 0.43–0.97) (Table 5).

**Table 5:** Alcohol drinking status odds ratio by different education level

Varibles	Odds ratio	95 % CI
Low education level		
Non drinkers vs. ex-drinkers	1.13	0.44–2.88
Non drinkers vs. current drinkers	1.73	0.84–3.58
ex-drinkers vs. current drinkers	1.53	0.62–3.80
Medium education level		
Non drinkers vs. ex-drinkers	0.72	0.44–1.18
Non drinkers vs. current drinkers	0.60	0.44–0.82
ex-drinkers vs. current drinkers	0.83	0.52–1.33
High education level		
Non drinkers vs. ex-drinkers	1.04	0.52–2.07
Non drinkers vs. current drinkers	0.65	0.43–0.97
ex-drinkers vs. current drinkers	0.62	0.33–1.16

## Discussion

The results of the cross–sectional study showed that that the overall prevalence of hypertension was 28.61 % among adult residents in Inner Mongolia Autonomous Region in 2014. The prevalence of hypertension was higher among Mongolian adults (31.46 %) than Han adults (27.47 %).

Our findings on the prevalence of hypertension among either Mongolian or Han adult residents were lower than that reported by other studies. The Study on Global Aging and Adult Health (SAGE) conducted by the World Health Organization (WHO) assembled nationally representative cohorts from 6 developing countries and found that the prevalence of hypertension varied from 23 to 52 % across different countries, and the prevalence among people aged 18 or older was 39 % in China [14]. Another study from China showed that the prevalence of hypertension for middle-aged and elderly hypertensive (35–74 years old) was 39.1 % in 2009 [15]. In another study from China, Li and colleagues reported the prevalence of hypertension among Mongolians aged 20 or older was 37.39 % between 2002 and 2003 [16]. The world health statistics 2015 showed that the overall prevalence of hypertension in adults aged 18 and over was around 22 % in 2014, and the prevalence was higher in male than female (24.0 % vs. 20.5 %). Across the WHO regions, the prevalence of hypertension was highest in Africa, where it was 30 % for both sexes combined as well as for men and women separately. The lowest prevalence of hypertension was in the WHO Region of the Americas at 20.8 % in male and 15.6 % in female. The prevalence of hypertension was higher in low-income countries compared to middle-income and high-income countries [17]. Indeed, reports from the United States survey and the WHO Multinational Monitoring Trends and Determinants in CVD Project indicated decrease trend in SBP and DBP from the 1980s to the 2000s in the whole population [6, 18]. The improvement in therapeutic control of high blood pressure among the general populations may account for the relative lower prevalence of hypertension in Mongolian and Han populations.

In this study, the adjusted prevalence of hypertension was higher in Mongolian (31.30 % vs. 26.45 %). The evaluation of ethnic differences in the clustering of cardiovascular disease risk factors (CRFs) of Xinjiang indicated that the prevalence was 55.9 % in Mongolian and 33.0 % in Han populations [19]. Prevalence was different in diverse populations and regions [20, 21]. A study in Yunnan showed that the hypertension prevalence varies among China’s ethnic groups, with 25 % in Hani minority and 64 % in Tibetan minority [22]. Different environmental exposures, ethnic-specific genetic susceptibility and the interactions between gene and environment may account for the different prevalence of hypertension [23, 24]. A study conducted in Inner Mongolia suggested that rs13306673 is a genetic factor for hypertension in the Han population but not in Mongolian population [25]. A further study between the prevalence of hypertension and ethnic specific genetic susceptibility is urgently needed to clarify the observation.

The conclusion that there were no significant difference between Mongolian and Han people in the prevalence of awareness, treatment and control of hypertension suggested that there was little difference of health resources between the two ethnicities. The awareness and treatment of hypertension were relatively high in Inner Mongolian of this study but only 43.58 % hypertensive people under treatment achieved BP control. Data from the National Health and Nutrition Examination Survey (NHANES) indicated that the awareness, treatment, and control of hypertension in 2008 was 80.7, 72.5 and 69.1 % respectively [6]. Among people aged 60 or over, the awareness and treatment were 78.37 and 92.76 % respectively, which were higher compared with the 30–39 years (*P* < 0.01). However, there was no significant difference in the proportion of hypertensive people with treatment and control compared with the 30–39 years (*P* > 0.05). The outcome may account for the fact that the older age is a key patient characteristic in treatment-resistant hypertension [26], and some studies also suggested ineffectiveness in our current treatment approach, which was largely based on the use of single drugs [27]. Hence, it is an important factor to improve hypertension control by enhancing the treatment effectiveness among individuals over 60 years old. The hypertension awareness, treatment, and control did not vary across ethnic groups. A study conducted in Inner Mongolia showed that the hypertension awareness, treatment and control were higher in Han than those in Mongolian [28]. Hypertension awareness, treatment and control in women were higher in men and it is consistent with other studies, which suggest that raising hypertension awareness, treatment and control are important for men. Hypertension awareness and control were lower in people with medium or high education level than those with low education. Generally speaking low education may influence access to care due to lack of knowledge of the sequelae of uncontrolled hypertension [29]. In our study, this may due to the samples with the medium or high education were more likely to be current smokers (21.45 %, 30.00 % vs. 12.50 %) and current alcohol drinkers (33.56 %, 57.50 % vs. 10.16 %).

Multiple logistic regression analysis showed that sex, age and BMI were significantly associated with hypertension prevalence in Mongolian. For Han people, there was no significant difference in men and women. The age, education, alcohol consumption, physical activity and BMI were significantly associated with hypertension. The prevalence of hypertension was lower in nondrinkers with medium or high education compared with current drinkers. Education was used as a surrogate for socioeconomic status in the study and similar social patterning of hypertension prevalence has been recently reported in some studies [27, 30]. In terms of risk factors, BMI was very important. In the US, from one-fifth to four-fifth of the increase in the prevalence of hypertension was attributed to the higher BMI [31]. A study conducted in Greece suggested greater BMI was significantly and directly associated with increased resting BP in both sexes [32]. In this study, 66.12 % of the hypertensive people were overweight or obese and weight loss should be considered highly important for the patients.

Limitations of our study are mainly those in epidemiological studies in general. Even though our analyses were adjusted for smoking and alcohol intake and other life styles, residual confounding due to lifestyle factors cannot be excluded. Furthermore, population may change their habits of drinking, smoking, physical activity when they got hypertension, which may result in selection bias. A study showed that the Chinese were less physically active overall, but they become much more physically active once they got cardiovascular disease [20]. In addition, the estimates of hypertension in this study, based on personal disease history or one time of blood pressure measure may overestimate the true prevalence of hypertension. In this study, the treatment of hypertension may have been underestimated because the definitions do not include individuals who have been diagnosed as hypertension but are controlling it by improving lifestyle alone.

## Conclusions

In conclusion, despite universal access to healthcare, Mongolian and Han groups living in Inner Mongolia differed in their prevalence of hypertension. These findings indicate that future hypertension efforts should develop ethnic specific strategies for preventing hypertension in Inner Mongolian. Further study on the association in the two ethnic groups’ underlying genetic factors is needed. Effective treatment and prevention measures are urgently needed to the high-risk urban populations and this will have a profound impact on public health.
